# The Field of Science

**Published:** 1893-11-04

**Authors:** 


### The Field of Science.

What the status, remuneration, and term of office should be of a medical officer of health, is a question lately under discussion in *Public Health*, which organ has constituted itself interpreter of various views on the subject. It would be a good thing were some definite settlement to be arrived at regarding this problem, which, in its present entanglement, is bringing many unpleasant consequences in its wake. As things now stand, the scale of payment of a medical officer of health is for the most part at so low a figure, that to increase ordinary means of subsistence, many have to look outside their own particular domain. Thus disagreeables have arisen from workers in the preventive department of the profession poaching on the preserves of those who practice medicine in its curative branch, and consequently there are often serious difficulties in the relations between the two branches of the profession.

At the same time, what is to be expected when a medical officer of health is offered the meagre stipend of £200 a year? It stands to reason he will wish to increase his income, and until more definite limits are assigned it is not to be wondered at that he has extended his sphere of action into territories of which others have a more rightful proprietorship. The consensus of public opinion is undoubtedly in favour of a complete severance of the two branches of the profession but the difficulty in bringing this about turns more especially on the question of salary. One plan, as a possible solution to this, has been suggested in the scheme to join several neighbouring districts together, wherein the work would not be too severe for one officer, and whereby his income would be substantially raised. By this means also not only would the income be raised, but by having more to occupy his time a medical officer of health need not look beyond his own department for means of necessary support.

We are gently reminded, in the pages of a Northern journal, that there are features in which the civilisation of the present century is inferior to that of the less enlightened days of our forefathers. It appears that the danger of using lead pipes for the conveyance of water into houses was not only recognised, but acted on in ancient Rome, and that a law actually prohibiting this was strenuously enforced. The paragraph on this subject to which we are making reference, is accompanied by the report of a carefully attested analysis of water which has been thus conveyed through lead service pipes, and which goes far to substantiate the writer's timely warning of the risk we run in continuing to use a poisonous medium for this purpose.

The Morgue is soon to serve an additional purpose, as it is proposed to place it in immediate connection with a medico-legal institute which is being contemplated for the Parisian capital. This new institute will include in its plans a toxicological laboratory, a work-room, a lecture-room, library, and museum. Rumour has it that the underground passage with which the building is connected with the Morgue is no other than a transformed sewer, but this is mere detail.

